# First Permanent Molars

**Published:** 1888-07

**Authors:** C. S. Case

**Affiliations:** Jackson, Mich.


### First Permanent Molars.

BY DR. C. S. CASE, JACKSON, MICH.

*The following scheme was presented to obtain statistics relative to the First Permanent Molars.*

[Read before the Michigan State Dental Association, held at Ann Arbor, Mich., March 20, 1888.]

If there is one question more than another upon which there is a wide difference of opinion among leading dentists, it is relative to the treatment of the first permanent molars between the ages of 7 to 13 that are difficult to save.

Some advocate the filling of them in advanced stages of decay, and under very unfavorable influences, for reasons which have been so often discussed it were useless to enumerate them here. On the other hand there is an equal array of ability who advise their extraction, under like conditions, in time to allow the second molars to take their places without irregularity if possible, and especially in a crowded jaw and under influences that render the ultimate saving of them improbable.

A year ago we listened to a short talk upon this subject by Prof. Watling—in lieu of a paper and models which he unfortunately left at home, and which I hoped would be presented at this meeting. He did not advocate the wholesale extraction of the first permanent molars, or the wholesale filling of them, even when possible, but chose rather to be guided by the conditions of each individual case and that which would promise the greatest future good to the whole mouth.

The short discussion which followed was but a repetition of that which takes place in every convention when this question is presented—the leading men in the profession often advocating radical and diametrically opposing theories and methods.

It is not my purpose to use the valuable time of this meeting in a further discussion of the subject, but wholly with the view of respectfully submitting a method for consideration which is intended to enlist the general cooperation of the profession in the procurement of data, that it is hoped will in time put the subject upon a more scientific basis, for since men's opinions so widely differ, if we can ever arrive at a more universally accepted system of practice it must be established by the certain knowledge of conditions in a large number of cases, under different methods of treatment.

Soon after the convention I made a rough draft of a letter to be addressed to each member of the Society, asking them to bring models to this meeting. I also made an historical blank, to be filled out and appended to the models. I had them put into print with directions to leave the forms standing for future changes, and there it has rested till now.

I bring this matter before you with the hope that it will receive the sanction and authority of the Society, and that a committee be appointed to correct the blanks and make such other arrangements as will be necessary for the fulfillment of the scheme; an explanation of which will be found in the letter as follows:

"*Dear Doctor:*—The question, 'What shall be done with the first permanent molars at the ages of from seven to ten years whose conditions are such as to foreshadow early loss?' is one of so much inportance, and about which authorities so widely differ, we have decided to solicit your aid in an endeavor to collect data that will place the subject upon a more scientific basis.

You are asked to make plaster models, upper and lower (and if possible the facial expression), of every questionable case that comes under your hands. In those cases where you have decided to save the said molars you will please mark upon the model the size and shape of the fillings; but if you have thought best to extract them please replant them in the models in their proper position, or preserve them in dilute alcohol.

To these preparations you will append the enclosed blank, filled out, and bring or send to our next annual meeting, where they will be properly systematized and labelled for future comparison.

During each subsequent year you will endeavor to make models of the same cases, and also add to your list new ones.

It is believed that when sufficient time has elapsed so that comparisons can be made ranging over a number of years, our collection will prove of great value in establishing some system of treatment that will be more universally accepted as the correct one. Respectfully,

——————————."

The following is the blank to accompany the first model, and to be filled out with each subsequent model:

---

Name——————————

Address——————————

Age and Date when First Permanent Molars were filled or extracted——————————

Date of Model No. 1——————————Age.

" " " No. 2—————————— "

" " " No. 3—————————— "

" " " No. 4—————————— "

" " " No. 5—————————— "

---

*REMARKS.*

---

DISCUSSION.

*Dr. J. Taft:* There has been no systematic effort to determine the history and character of the first permanent molars.

There are differences of opinion on the subject. Some say all should be extracted. Others say that this should be done only in special cases. It is very desirable that we should know all that can be learned in regard to preserving them. A series of experiments extending over several years and carried on by persons interested would be very valuable and would settle many questions. I believe Dr. Case's idea is a very good one. The blank he has prepared contains the questions most necessary. It is not much but exact data that we wish, and that is what the blank purposes to get. This blank should be filled out and sent to some person who should have it in charge. The experiments should be followed up very soon after, and then each year for several years. Thus we might learn what changes in position the teeth undergo after the first molars. Sometimes one and again all change. The general rule is a matter of question. Is it desirable to leave a large space between the teeth? Sometimes the molars will lean toward each other, the teeth will strike each other at an angle so that there can be no good grinding nor thorough salivation. In what proportion do they come up square? That is another question which might be settled in this manner. What part does the third molar play? Does it always become a good masticating organ? What is the liability in these cases? It is necessary to know this. These and many other questions could be scientifically settled by a series of experiments carried on through a number of years. But who is to do it? The profession is too busy making dollars to follow up such work. They will not take the time it needs. The best and most expeditious way is to call for volunteers. If a dozen, half a dozen, or even three men follow it up, it would be of great service to the profession.

*Dr. C. S. Case:* The paper was not written to provoke discussion on the subject itself, but in order to get you to bring to our meetings models of various kinds, and, further, to set in motion a line of investigations. Will you do it? Or must we go on for years in the same old rut without any new ideas on the subject, without endeavoring to learn more concerning this important matter? Can we not appoint a committee to take charge of it?

*Dr. R. Douglass:* I would be in favor of such a committee and would suggest that the members of the proposed committee be young men. They will have a better chance of getting a longer series of experiments. They can take it up where we older men leave off.

*Dr. J. Taft:* The most advisable way would no doubt be to call for volunteers. It would scarcely be worth while for any one to take hold of the matter who does not expect to remain in one locality for some time. Let these consult with Dr. Case.

*Dr. C. S. Case:* Take the impression of the mouth and even of the face, when practicable, of patients coming under your care.

